# Synthesis, Characterization, and *In V* *ivo* Toxicological Evaluation of Copper (II) Oxide Containing Herbometallic Siddha Nanocomplex “Thamira Parpam”

**DOI:** 10.3389/fbioe.2022.849441

**Published:** 2022-04-11

**Authors:** Parameswari Royapuram Parthasarathy, Verasundaram M. Manikandamathavan, Chandranayagam Chandronitha, Hannah R. Vasanthi, Vasanth Kumar Mohan, Venkataramanan Vijayakumar, Rajeshkumar Shanmugam, Saravanan Sekaran, Balachandran Unni Nair, Duraipandian Chamundeeswari, Sadras Panchatcharam Thyagarajan

**Affiliations:** ^1^ Centre for Transdisciplinary Research, Department of Pharmacology, Saveetha Dental College and Hospitals, SIMATS, Chennai, India; ^2^ Herbal and Indian Medicine Research Laboratory, Sri Ramachandra University, Chennai, India; ^3^ Chemical Laboratory, Central Leather Research Institute, Chennai, India; ^4^ Department of Chemistry, Thiruvalluvar College, Tamilnadu, India; ^5^ Department of Biotechnology, School of Lifesciences, Pondicherry University, Tamil Nadu, India; ^6^ Avinashilingam Institute for Home Science and Higher Education for Women Coimbatore, Tamil Nadu, India

**Keywords:** copper oxide, Siddha, herbometallic, Thamira parpam, nanocomplex, toxicity

## Abstract

“Thamira parpam” (TP), a copper-based herbometallic oxide (copper (II) oxide) nanodrug has been used in Siddha medicine for centuries because of its anti-ulcerogenic property. However, the physicochemical properties and *in vivo* toxicity of TP still remain elusive. Rigorous clinical translation requires deciphering these vital properties. We have synthesized TP following a gold standard protocol in the traditional Siddha methodology. We assessed the size, phase, elemental constituents, and thermal stability of TP by SEM and TEM, XRD, EPR, and EDAX analyses, respectively. The results depicted the conversion of metallic copper into copper (II) oxide in the final stages of TP preparation and exhibited nanodimensions ranging between 10 and 50 nm. The XPS spectra revealed the presence of oxygen-deficient state and a carbonaceous coating was found on the surface of TP using TEM analysis. *In vivo* safety was studied in rat toxicity models by adopting OECD guidelines. Body weight changes, feed, and water intake were unaltered upon TP administration. Hematological, biochemical profiling, and histopathological findings also suggested its nontoxic nature with no abnormalities in major organs and its functions. Interestingly, we found that the metal toxicity could have been subdued because of the carbonaceous coating around the nanoparticle copper (II) oxide, confirming that the drug is safe at a low dose. Overall, our study has enlightened the safety of TP supporting the use of Siddha formulations.

## 1 Introduction

Metal nanoparticles have gained significant interest over the past decade because of their unique properties and biomedical applications (Nations et al., 2015; Shnoudeh et al., 2019; Cid and Simal-Gandara, 2020; Matur et al., 2020; Umapathi et al., 2020; Patel et al., 2021; Umapathi et al., 2021; Kumawat et al., 2022). The Indian systems of medicine, such as Ayurveda and Siddha, considered the greatest medical heritage have developed and practiced the use of metal oxide nanoparticles as medicines that use metals and herbs as their integral parts (Bhowmick et al., 2009). These metal-based nanoparticles possess unique properties and features and have created a huge impact on modern medicine (Fricker, 2007). The herbometallic combination, which is in the form of a nanoparticle termed as *Bhasmas or Parpams (ash)* in the alternative medicine systems, has shown to impart targeted and effective therapeutics against various chronic and long-term diseases/disorders (Nagarajan et al., 2014). Moreover, these drugs are found to be effective at very low concentrations and are advised to be taken along with suitable adjuvants such as milk, ghee, or honey (Sudha et al., 2009).

Metal oxide nanoparticles have been prepared by using several synthesis techniques and are considered to play a critical role in imparting uniqueness for the final nanoparticle and thereby having an impact on its medicinal properties (Tanna et al., 2015; Grigore et al., 2016; Chaudhary et al., 2019; Chouke et al., 2019; Alyamani et al., 2021). The classical texts of Ayurveda and Siddha claim that it is the distinctive and holistic method of preparation that confers significance to its metal oxide nanodrugs (Subbarayappa, 1997; Sheikh, 2004; Nagarajan et al., 2012). The preparation method of these herbometallic oxide nanoparticles involves elaborate and iterative processing steps of annealing, detoxification, and calcination. It is believed that during the synthesis process, the metals from their raw state, in combination with the herbs, get converted to their respective oxides, thereby eliminating the metal toxicity and imparting medicinal properties (Subbarayappa, 1997). However, metal concentrations exceeding physiological levels could be toxic since they are able to generate free radicals that result in lipid peroxidation and the depletion of the sulfhydryl groups (Florea and Busselberg, 2008). Therefore, an intensive investigation on the method of preparation, its standardization, and especially the safety and toxicity of herbometallic preparations has become the need of the hour not only for it to be accepted by modern medicine systems but also for a better understanding of its applications on health effects.

Among the metal oxide nanoparticles, copper oxide nanoparticles are found to possess excellent medicinal properties. Copper oxide nanostructures possess multifaceted applications in the field of biomedicine. It is found to exhibit anticancer (Pillai et al., 2022), wound healing (Ruddaraju et al., 2021), antibacterial properties (Bezza et al., 2020), used as coating in metallic dental implants (Vergara-Llanos et al., 2021), possess anti-inflammatory, antitumor property, and several other biomedical applications that are extensively reviewed by Verma and Kumar, 2019). “Thamira parpam” (TP), a copper-containing herbometallic oxide nanocomplex, has been in human use since antiquity for the management of ulcers. TP is known as “Gunma kalan” in Siddha literature which means that the drug is effective in healing ulcers. The anti-ulcer efficacy of TP has already been reported in rats from our laboratory (Parameswari et al., 2010; Vasanthkumar et al., 2010). The purified form of copper oxide is used in the treatment and management of obesity, tuberculosis, cough, asthma, skin diseases, and obesity (Bhavyasree and Xavier, 2020). As most nanodrugs under the classification of alternative medicine demand details of adverse effects, the present study is an attempt to determine the safety and toxicity profile of the copper oxide–based TP. Though similar studies were carried out for Ayurvedic bhasmas, namely, Tamra bhasma, Naga bhasma, and, Jasada bhasma, scientific reports on characterization and standardization of Siddha preparations are seldom carried out (Wadekar, 2005; Bhowmick et al., 2009; Nagarajan et al., 2014). To our knowledge, this is the first report in ancient Siddha medicine research that has comprehensively carried out the chemical characterization of a Siddha herbometallic oxide preparation using modern techniques.

## 2 Materials and Methods

### 2.1 Drugs and Chemicals

Copper turnings (99.9% pure) were purchased from the Central drug house, Chennai, Tamil Nadu, India. The whole plant of *Tamarindus indicus*, *Aloe vera*, *Alternanthera sessilis*, *Aristolochia bracteolata*, and roots of *Alangium salviifolium* were collected from Tirunelveli, Tamil Nadu, India, by Mr V. Chelladurai, Retd. Research officer, Central Council of Research in Siddha and Ayurveda (CCRAS) and were authenticated by Professor Jayaraman, Plant anatomist, Plant Anatomy Research Center, Tambaram, Chennai, India. The specimen vouchers (SRU/2010/014, SRU/2010/015, SRU/2010/016, SRU/2010/017, and SRU/2010/018) were preserved in the Center for Indian Systems of Medicine, quality assurance and standardization, Sri Ramachandra Institute of Higher Education and Research (DU), Chennai, Tamil Nadu, India. The standard copper oxide (99.9% pure) used for comparative studies was purchased from Sigma Aldrich, United States. All the other chemicals used were of analytical grade.

### 2.2 Preparation of Thamira Parpam

#### 2.2.1 Purification of Copper

Copper sheets (1.75 kg) were preferred as the source of pure metallic copper (purity >99.9%). The copper sheets were cut into small pieces (approximately 5 mm × 5 mm) and heated until red-hot using a manual blower. At the red-hot state, the sheets were quenched in *Dolichosbi florus* (horse gram) decoction (1 L) and taken out when cooled to room temperature. The process was repeated six times with a new horse gram decoction (suthi process) each time. Again, the same process was repeated seven times each with *Tamarindus indica* leaf juice, *Aloe vera* juice, *Alternanthera sessilis* juice, and sour butter milk. The time taken for heating the copper sheets and subsequent quenching in herbal juices seven times was around 7–10 h for each process. Then, the copper sheets were dried under sunlight and stored in a covered mud plate for further processes.

### 2.3 Process of Maaranam

The purified copper sheets (1 kg) were covered with a paste of rock salt (1 kg) and lemon juice. After drying, the same was kept in a mud plate and covered with another mud plate of the same size. It was sealed with seven layers of mud-smeared cotton cloth and allowed to dry. Then, it was subjected to calcination (*“Pudam”—Traditional Siddha method of preparing herbometallic drugs*) using cow dung cakes (50 no. of average weight of 100 g each). The product obtained after calcination is stored for further processes. The time taken for the process of Maaranam was around 4–5 days.

### 2.4 Calcination of Copper

The purified copper (1 kg) after the completion of Maaranam was ground with juice (500 ml) of *Aristolochia bracteolata* for 6 h and made as to tablets (Approx. 100 g). The same was dried in sunlight. After complete drying, the tablets were kept in a mud plate and covered with a mud plate of the same size. This was sealed with seven layers of mud-smeared cotton cloth and allowed to dry. Then, it was subjected to calcination using cow dung cakes (100) no. of average weight of 100 g each). The product, thus, obtained is finely powdered, and the aforementioned process is repeated with fresh juice of *Aristolochia bracteolata.* The time taken for each calcination step was around 6–7 days. The calcination was performed 15 times with the juice of *Aristolochia bracteolata* followed by 10 times of calcination with *Alangium salviifolium* decoction. The final product in the form of the pellets was taken out of the earthen pot and powdered. The powdered material was finely sieved and packed in airtight containers.

### 2.5 Physical Measurement and Chemical Characterization

Thermogravimetric analysis was carried out using the Q50 TGA instrument. Thermogram was run from 30 to 800°C in the presence of nitrogen gas and a heating rate of 20 C/min. X-ray photoelectron spectroscopy (XPS) was carried out using the KRATOS AXIS 165 X-ray photoelectron spectrometer to know the correct mechanism of formation of the CuO phase in TP. Information on crystalline character and phase purity was obtained from XPERT PRO, PANalytical XRD using a Cu–Kα radiation. The EPR spectrum was recorded using a Bruker EMX 10/2.7 X-Band EPR spectrometer operating at the X-band and using 100-kHz magnetic field modulation. The chemical composition of the sample was determined by EDAX attached to high-resolution SEM (FEI Quanta 200 FEG). The size of the nanoparticles was characterized using a transmission electron microscope (TEM) analyzed with Philips Tecnai 10 (Philips, Netherlands).

### 2.6 Toxicity Studies—Experimental Animals and Study Design

#### 2.6.1 Animals

Sprague–Dawley (SD) rats of male and female sex weighing 150–200 g were obtained from the Centre for Toxicology and Developmental research (CEFT), Sri Ramachandra University, Chennai, India. The animals were housed in groups of five in polypropylene cages. All the animals were kept in one room with no other species being housed in the same room. The room was well-ventilated with 100% fresh air. Temperature and relative humidity were maintained, monitored, and recorded twice a day and found in the range of 19.22–22.75°C and 045.6–066.0%, respectively. Then, 12–15 air changes/h were maintained in animal confinements. The animals were maintained in 12 h light artificial photoperiod and 12 h dark. The animals had free access to pelleted feed of standard composition containing all macro- and micro-nutrients. Aquaguard (on-line water filter-cum- purifier) water was provided *ad libitum*. The animals used in the present study were housed and cared in accordance with the CPSCEA guidelines, India and Institutional Animal Ethics Committee (IAEC/SRU/65/2006) of Sri Ramachandra University, Chennai, India, approved the study.

### 2.7 Selection of Drug Dosage

For the acute toxicity study, a maximum dose of 2000 mg/kg b wt (as per OECD guidelines 423) was administered as single exposure. The test drug dose for a 28-day repeated oral toxicity study was selected based on the human dose used by Siddha practitioners (conversion to rat dose was performed on the basis of the body surface area of human to rat). A mid-dose (20 mg/kg/day) was derived as mentioned before; from this point, a lower dose of one-fold (10 mg/kg/day) and a higher dose of two-fold (40 mg/kg/day) were selected for the 28-day repeated oral toxicity study.

### 2.8 Acute Toxicity

Healthy SD rats of female sex were randomly divided into two groups (n = 3/group). Group I (control group) received 40% honey orally and group II was orally treated with Thamira Parpam of 2000 mg/kg b wt suspended in 40% honey as a single dose as per OECD guidelines 423, Acute Toxic Class Method (OECD, 2001). After drug dosing, the animals were observed for clinical signs of toxicity such as ataxia, convulsion, exophthalmia, lacrimation, oral/nasal discharges, gait, piloerection, polyuria, and rough coat at 30 min, 1, 2, 4, and 8 h. Furthermore, the animals were observed daily for mortality, behavioral abnormalities, body weight changes, and other mentioned toxicity symptoms for a period of 14 days after drug treatment.

### 2.9 28-Day Repeated Oral Toxicity

The method was performed according to OECD Guidelines 407 (repeated 28 days oral toxicity study in rodents, 2008). Healthy male and female SD rats were randomly divided into five groups by sex (n = 5/group). Group I animals served as the normal control; group II received 40% honey which served as the vehicle control. Group III, IV, and V served as treatment groups TP with doses of 10, 20, and 40 mg/kg/day respectively, administered for 28 consecutive days. The animals were monitored daily for mortality, morbidity, clinical signs of toxicity (mentioned as in acute toxicity study), functional observations (motor activity, grip strength, visual response, proprioceptive response, and auditory response), feed consumption, and body weight changes during the experimental period.

### 2.10 Hematological Analysis

Following 28 days of treatment, animals were starved overnight (∼18 h), but allowed access to water *ad libitum*. The animals were anesthetized and 0.5 ml blood was collected using sterile capillaries by retro-orbital puncture in tubes containing ethylenediaminetetraacetic acid (EDTA) for hematological analysis. The blood samples were used for the analysis of total and differential WBC count, total erythrocyte count (RBC), hemoglobin (Hb), hematocrit (HCT), platelet count (PLT), plateletcrit (PCT), mean corpuscular volume (MCV), mean corpuscular hemoglobin (MCH), mean corpuscular hemoglobin concentration (MCHC), and red blood cell distribution width (Rdw-Cv). Hematological analysis was performed using a fully automated hematology analyzer (PE6000, Aspen Diagnostics Pvt Ltd.).

### 2.11 Biochemical Analysis

The blood samples were collected using sterile capillaries by retro-orbital puncture in tubes containing trisodium citrate for clinical biochemistry analysis. Plasma was separated and stored at −40 ± 2°C prior to biochemical analyses. To identify the toxicity-related changes due to the dosing of the test drug, the various plasma enzymes, namely, serum glutamic oxaloacetic transaminase (SGOT), serum glutamic pyruvic transaminase (SGPT), alkaline phosphatase (ALP), acid phosphatase (ACP), γ-glutamyl transferase (GGT), and lactate dehydrogenase (LDH) were analyzed. Similarly, plasma metabolites such as blood glucose, bilirubin, cholesterol, triglycerides, protein, albumin, urea, and creatinine were also determined by the Biosystem kit using a fully automated biochemical analyzer (A15, Biosystems).

### 2.12 Necropsy and Organ Weights

All the tested animals were euthanized and subjected to gross necropsy, and the findings were recorded. After detailed gross necropsy examination, the organs such as eyes, brain, heart, lungs, stomach, liver, spleen, kidneys, adrenals, and ovaries/testes were collected from each animal, and the weights were recorded immediately using a top-loading electronic weighing machine (GP3202, Sartorius).

### 2.13 Histopathology

The tissue from the aforementioned organs was collected from all the animals and preserved in 10% buffered neutral formalin. They were sliced adequately wherever necessary. After a minimum of 24 h fixation, the samples were processed by conventional paraffin block methods and 6-μm paraffin sections were stained with hematoxylin and eosin. They were examined under a light microscope. All deviations from normal histology were recorded and compared with the corresponding controls.

### 2.14 Statistical Analysis

Statistical analysis was carried out for data collected on body weight, food, water intake, hematological, and biochemical analysis. Data were expressed as mean ± SEM. One-way ANOVA followed by Tukey’s post hoc test was used to compare the mean difference between the control with the vehicle group and vehicle with drug-treated groups using GraphPad prism 7.0. ‘P’ value of <0.05 was considered to be significant.

## 3 Results

### 3.1 Photoemission Electron Spectroscopy (XPS)

**FIGURE 1:**
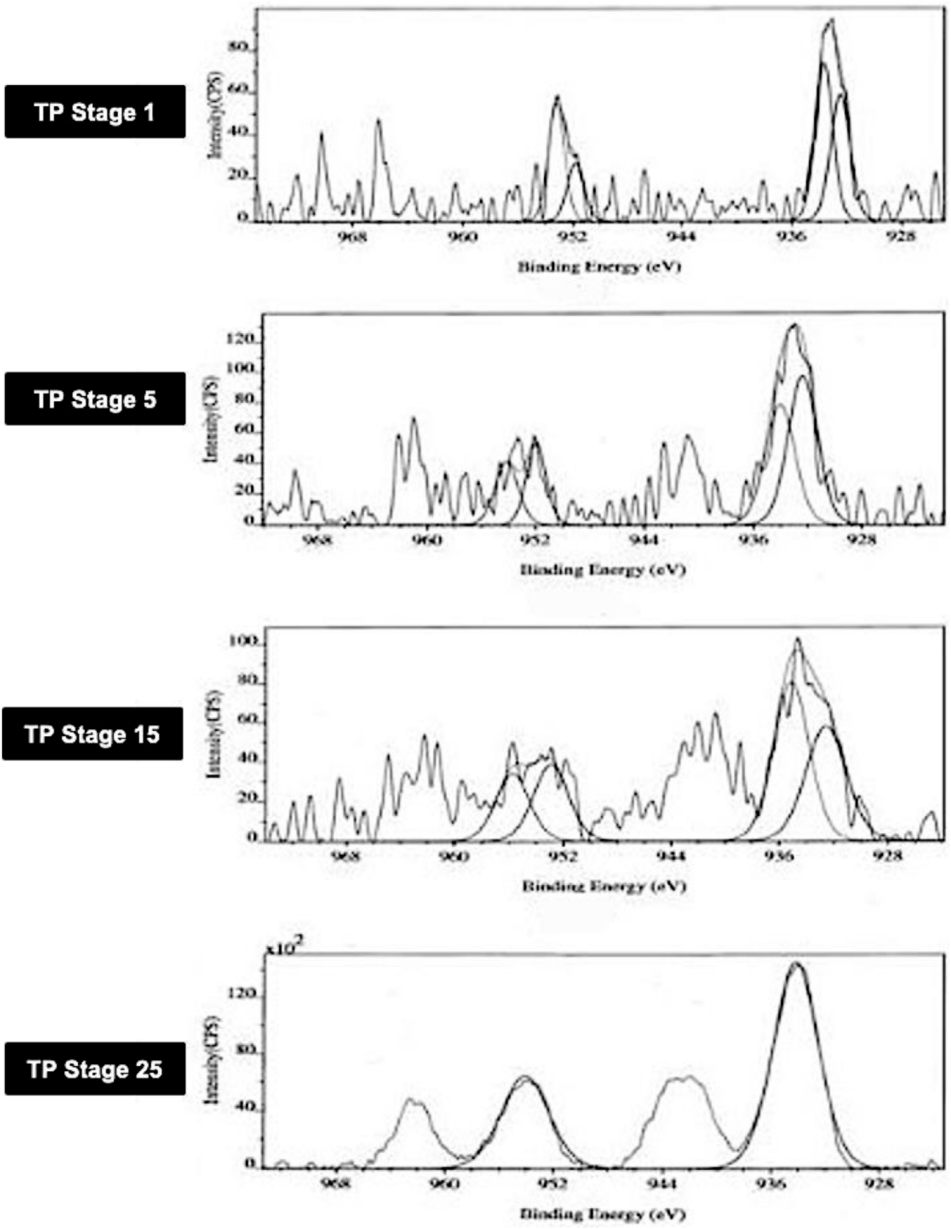
Photoemission electron (XPS) spectra of different stages Thamira parpam prepared using the traditional ancient method, showing peak positions of the Cu2p_3/2_ and Cu2p_1/2_ peak of CuO **(A)** Stage-1 TP; **(B)** Stage-5 **(C)** Stage-15 TP **(D)** Stage-25 TP.

The XPS spectra of TP collected at four distinctive stages of its preparation were analyzed in order to know the surface state of the drug sample. The core level spectra of the different stages of the samples marked as TP-1, TP-5, TP-15, and TP-25 are shown in Figure 1. The peak positions of Cu2p_3/2_ and Cu2p_1/2_ in XPS spectra of TP are also shown. The Cu2p_3/2_ peak is generally broad (3.4 eV) with a binding energy of 933.2 ± 0.2 eV and is accompanied by a satellite peak on the high-binding energy side at about 944 eV. This satellite peak is characteristic of materials having a d9 configuration in the ground state corresponding to CuO. The d9 electronic configuration typical of Cu (II) derivatives promotes d–d transitions. The spectrum of Cu_2_O, on the other hand, has only one peak at 932.4 ± 0.2 eV, which is significantly narrower (1.9 eV).

In TP-1, the binding energies of Cu2p_3/2_ and Cu2p_1/2_ observed at 933.5 ± 0.2 eV and 951.8 ± 0.2 eV corresponds to CuO and are characteristic of pure CuO as per previous published reports and database (Wadekar, 2005; NIST database, 2000; Potbhare et al., 2019). Similarly, the intermittent samples TP-5 and TP-15 exhibited binding energies of Cu2p_3/2_ and Cu2p_1/2_ which appeared at 932.6 and 952.9 eV and 932.3 and 951.9 eV, respectively. In addition, the intermittent sample TP-15 also showed two extra peaks which were observed at 935 ± 0.2 eV and 955 ± 0.2 eV. This sample exhibited signals at 529.2 ± 0.3 and 530.2 ± 0.2 eV, which are characteristic of oxygen and adsorbed oxygen for CuO and Cu_2_O, respectively.^14^ In the final product (TP-25), the binding energies of Cu2p_3/2_ and Cu2p_1/2_ showed the presence of CuO; the peak due to Cu_2_O was absent in this case, indicating that the product at this stage was completely CuO.

### 3.2 Thermogravimetric Analysis

**FIGURE 2:**
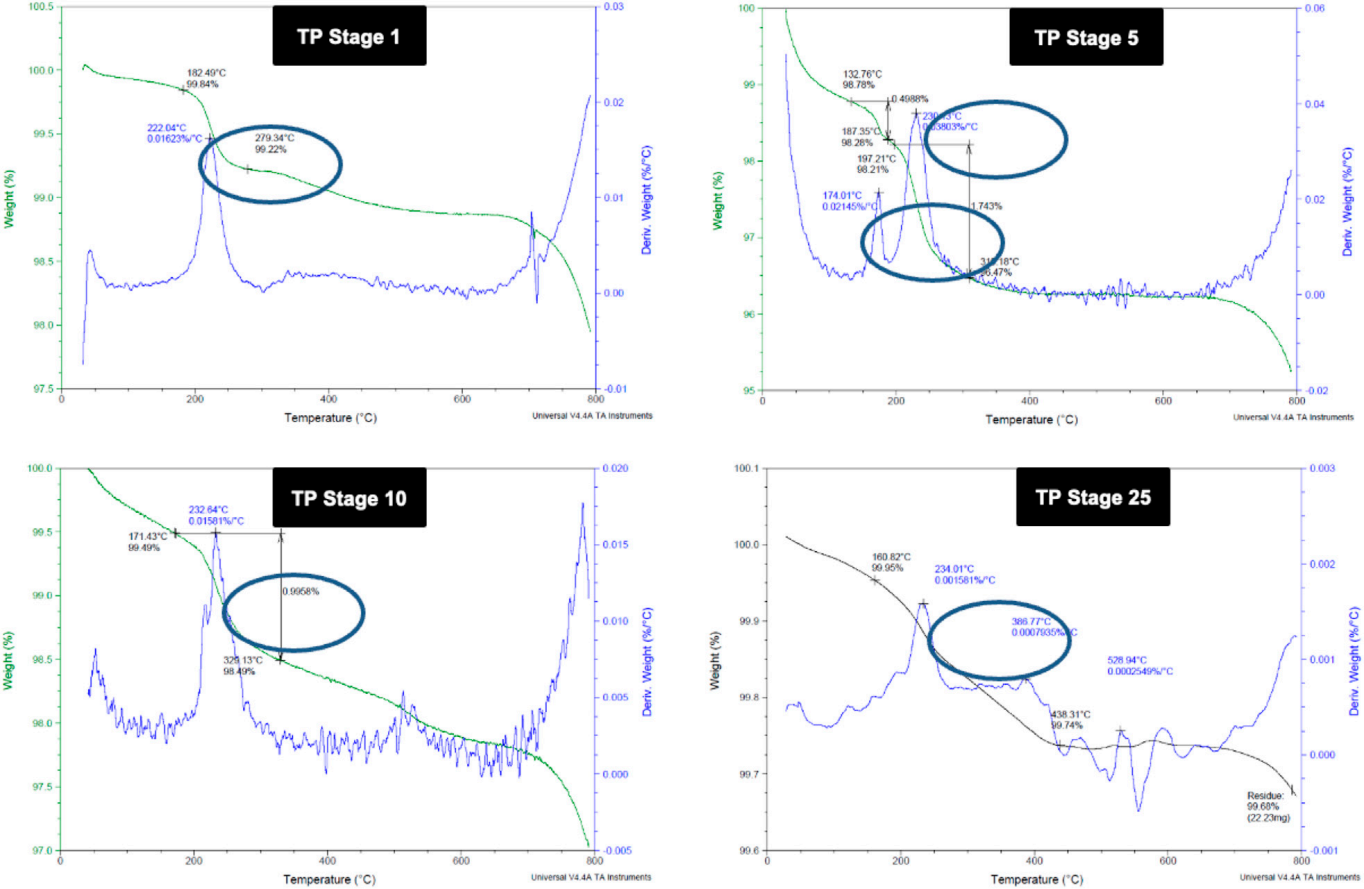
Thermogravimetric analysis (TGA) of different stages of Thamira parpam prepared by the traditional ancient method **(A)** Stage-1 TP; **(B)** Stage-5 **(C)** Stage-10 TP **(D)** Stage-25 TP.

Thermo gravimetric analysis of the TP sample at different stages of preparation was carried out to find the decomposition pattern (Figure 2). Thermal decomposition occurred over a temperature around 182°C for sample TP-1 with a weight loss of about 0.62%, whereas two weight loss regions were observed for sample TP-5 with the onset at 132°C and 197°C temperatures and weight losses of 0.49 and 1.74%, respectively. The 15th stage TP again exhibited a weight loss region with the onset temperature at 167°C and a weight loss of 0.47%. No further weight loss was observed from stages 18 to 25, indicating the conversion of copper to its stable copper oxide.

### 3.3 Electron-Paramagnetic Resonance

**FIGURE 3:**
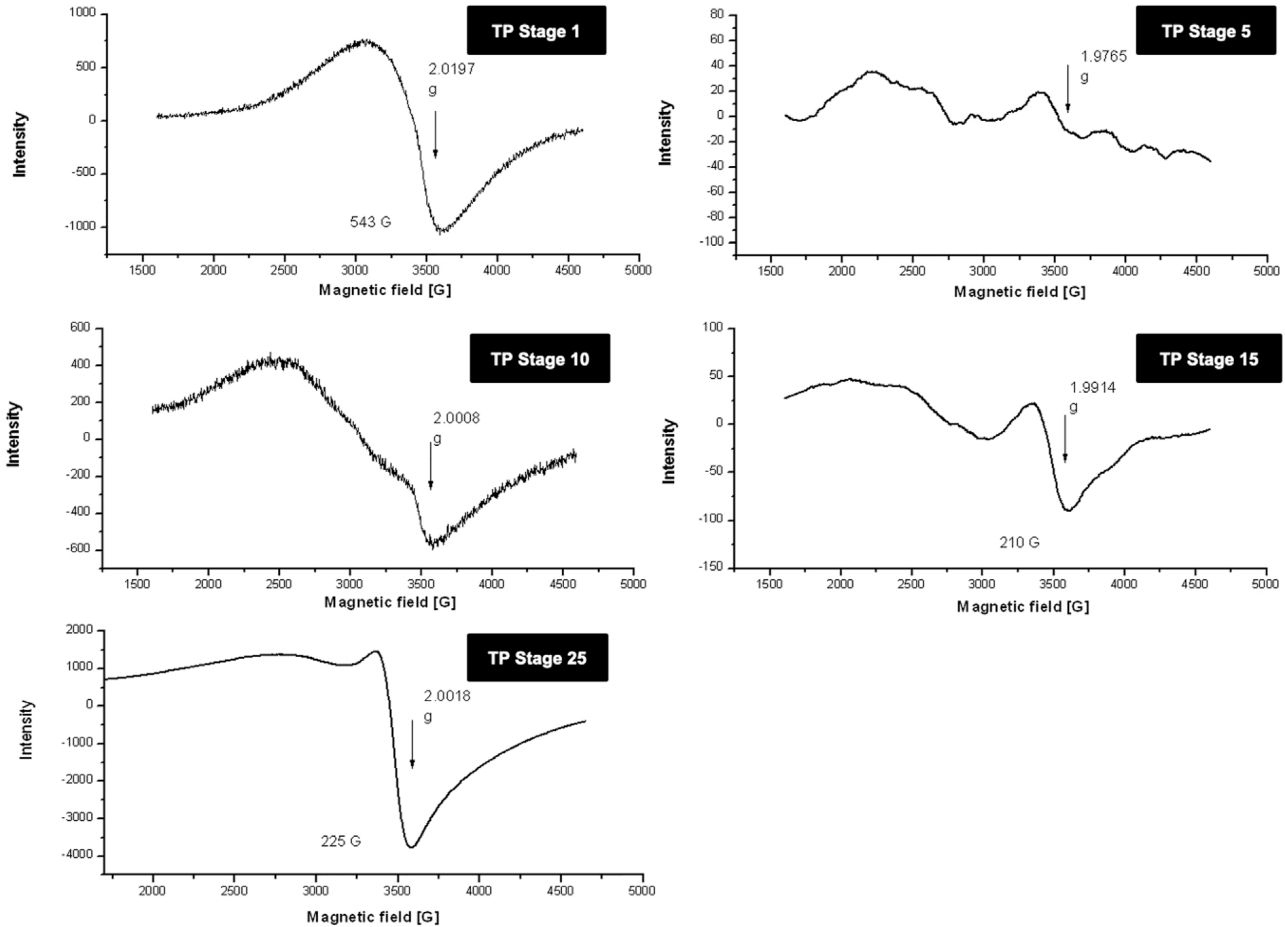
Room temperature electron paramagnetic resonance (EPR) spectra of various stages of Thamira parpam prepared by the traditional ancient method in comparison to the laboratory method of TP preparation. **(A)** Stage-1 TP; **(B)** Stage-5 **(C)** Stage-10 TP **(D)** Stage-15 TP; **(E)** Stage-25 TP.

Room temperature and low temperature quantitative EPR was carried out to determine the oxidation state of copper in various stages of traditional TP preparation. Figure 3 shows the room **t**emperature EPR spectrum of various stages of the pudam samples. TP-1 showed a broad EPR signal with a g value of 2.0197, typical of CuO. The EPR signal of TP-5 was very weak, indicating a reduction of Cu (II) to Cu (I). The EPR signal near g ≈ 2 displayed by TP-10 also indicates the presence of the Cu (I) complex along with Cu (II). The repeated calcination method that involves treatment of copper metal with aqueous juices of Aristolochia and Alangium during TP preparation perhaps would be the reason for the sudden shift in the EPR pattern. The spectra obtained at the 15th stage (TP-15) again clearly showed the presence of some Cu (I) complexes with CuO. The spectrum of the sample obtained at the 25th stage (Final TP) is more prominent in matching with the spectra of CuO. However, the signal, in this case, was much sharper than that obtained for pure CuO.

**FIGURE 4:**
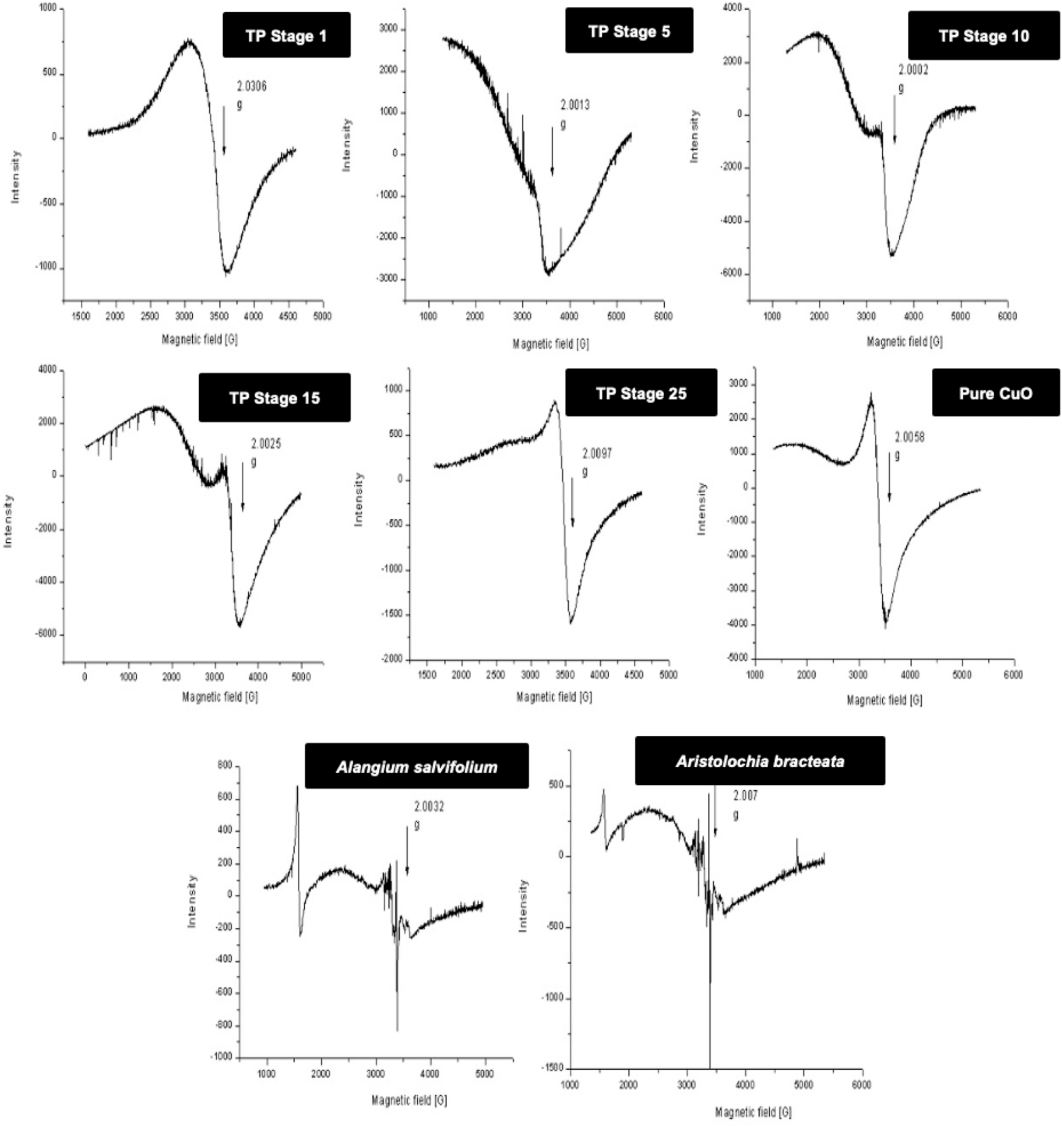
Low-temperature (77 K) electron paramagnetic resonance (EPR) spectra of various stages Thamira parpam prepared by the traditional ancient method in comparison to the laboratory method of TP preparation. **(A)** Stage-1 TP; **(B)** Stage-5 **(C)** Stage-10 TP **(D)** Stage-15 TP; **(E)** Stage-25 TP; **(F)** CuO **(G)** Alangium salviifolium; **(H)** Aristolochia bracteata.

In order to obtain more insight, 77-K (low temperature) EPR spectra were also collected for the samples (Figure 4). These results once again confirmed that the samples obtained at the intermediate stages do contain some copper (I) complexes along with CuO. The spectrum of the sample obtained at the final 25th stage was much narrower, suggesting some morphological changes in CuO present in TP due to spin–spin interaction and was indicative of pure CuO. The EPR spectrum of lyophilized plant juices was also analyzed. Organic molecules possess the nature of burning out at temperatures >400°C. Interestingly, the extracts of both the plant juices showed a sharp signal at g ≈ 2, indicating the presence of organic moieties.

### 3.4 X-Ray Diffraction Pattern

**FIGURE 5:**
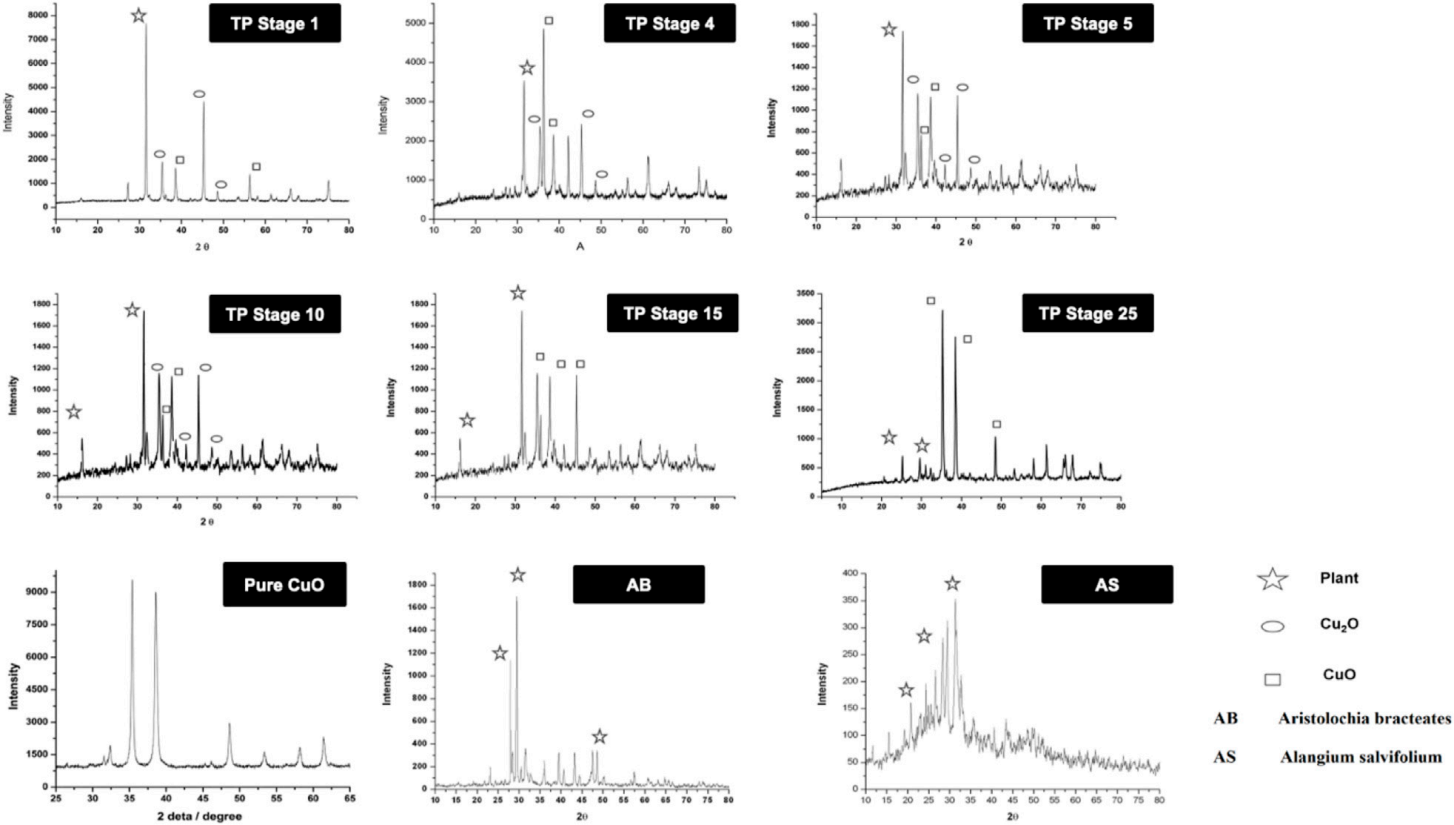
X-ray diffraction pattern (XRD) of various stages of Thamira parpam prepared by the traditional ancient method in comparison to the laboratory method of TP preparation. **(A)** Stage-1 TP; **(B)** Stage-5 **(C)** Stage-10 TP **(D)** Stage-15 TP; **(E)** Stage-25 TP; **(F)** CuO; **(G)** Aristolochia bracteolata; **(H)** Alangium salviifolium.

The XRD of the powdered sample of traditional TP obtained at various stages, namely, TP-1, TP-5, TP-10, TP-15, and TP-25 was recorded for phase identification. A typical XRD pattern of lyophilized Aristolochia and Alangium plant juices and pure CuO was also documented and is shown in Figure 5. The XRD pattern of pure CuO annealed at 400°C exhibited a monoclinic structure. Although the XRD pattern of Aristolochia revealed a sharp crystalline structure, the diffraction pattern of Alangium appeared slightly amorphous. The phase intensity of CuO in TP-1 was very low along with some other phases. The XRD of TP at the 10th stage clearly indicated the presence of CuO, besides some extra peaks, which might have been derived from the herbal sources. The diffraction pattern of TP-15 clearly showed the presence of Cu_2_O phase at 2Ө = 42.3, 61.6, 73.7, corresponding to the (200), (220), and (311) planes, along with the CuO phase at 2Ө = 35.36, 48.71 with (11‾1) and (20‾2) which is in strong agreement with JCPDS-ICDD ref. 004–0836. Aristolochia clearly showed the presence of intense peak at 2θ = 26, 28 along with the less intense peaks 23, 36, 38, 43, and 47. Alangium exhibited a broad XRD pattern from 2θ = 18 to 37. Both the plants showed peaks around 2θ = 30, which might be due to the carbonaceous material from the plant. From the results, it can be seen that the peak corresponding to the plants decreases and the peak due to metal oxide increases during each step of pudam methodology. Hence, from the results of XRD it was confirmed that copper (I) oxide had formed in the intermediate stages which got converted into copper oxide during the final stages. The final stage TP (TP-25) confirms the absence of crystalline structure. A comparative analysis of XRD results between TP and pure CuO showed a similar peak pattern at identical positions; thereby confirming that the final product of TP contains CuO (JCPDS-ICDD ref. 004–0836).

### 3.5 Scanning Electron Microscope and Energy Dispersive X-Ray Analysis

**FIGURE 6:**
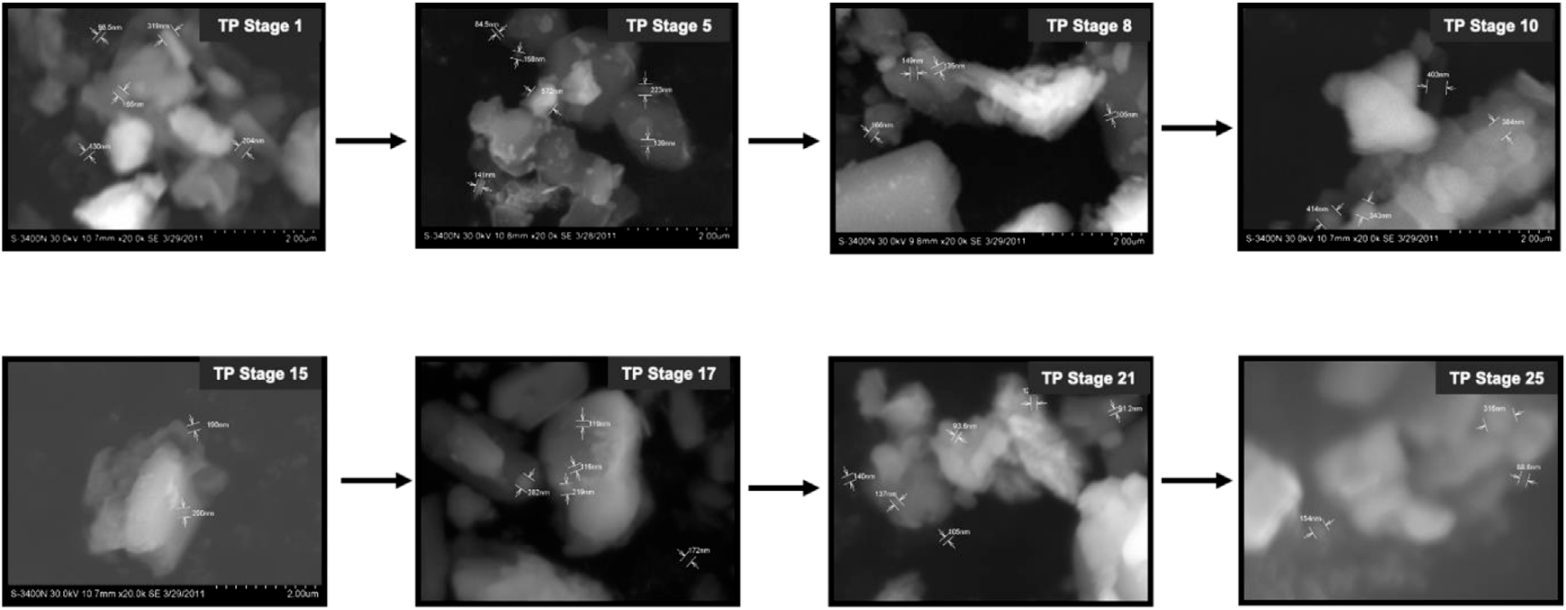
Scanning electron microscopic (SEM) images of various stages Thamira parpam prepared using the traditional ancient method.

**FIGURE 7:**
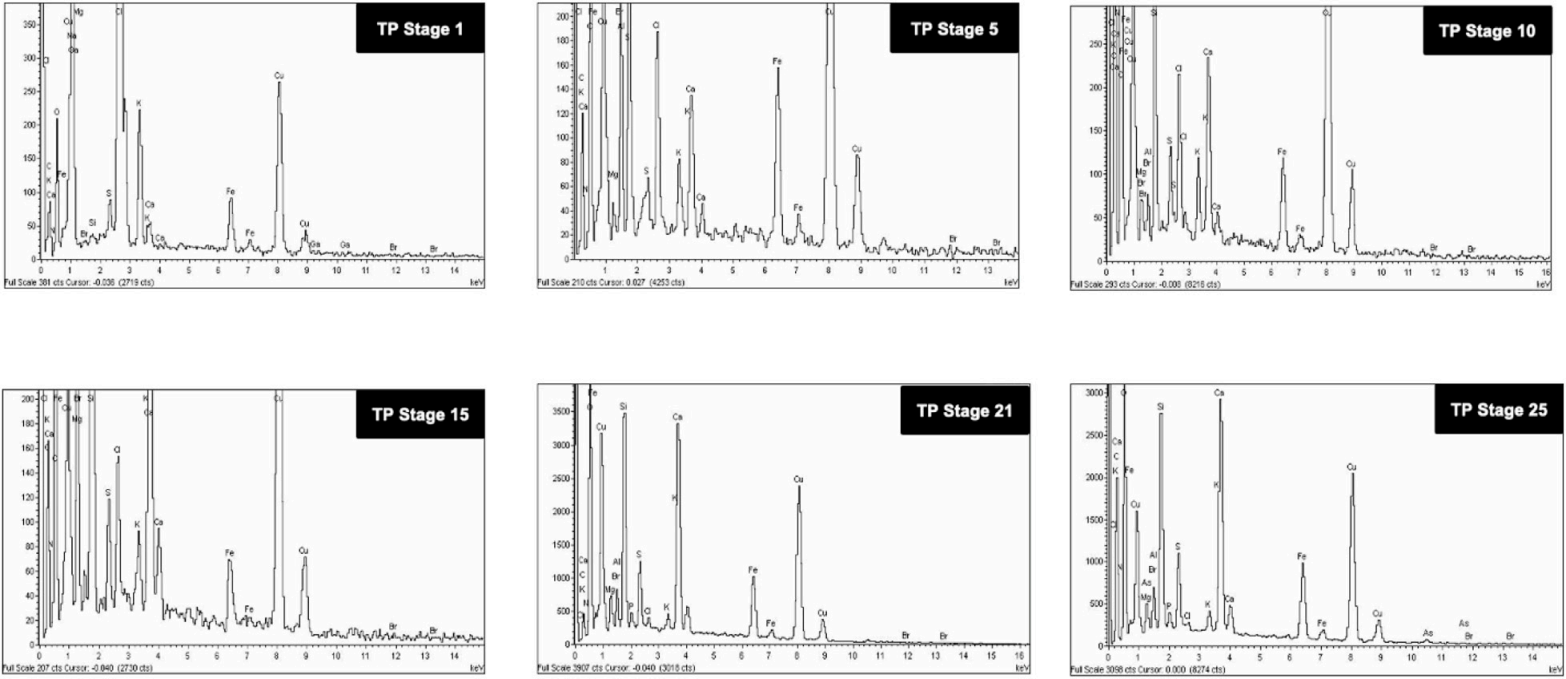
Energy dispersive X-ray analysis (EDAX) of various stages of Thamira parpam prepared using the traditional ancient method showing presence different metals and trace elements.

The SEM images of different stages of traditional TP have been displayed in Figure 6. It was quite evident from the images that initial stages of traditional TP (TP-1 to TP-15) have exhibited difference in size and agglomeration. The SEM image of final stage TP (TP-25) appeared with less agglomeration showing distinct morphology. The elemental compositions of traditional TP were analyzed by EDAX (Figure 7). All the samples showed the presence of significant amount of Fe, C, Na, and Cl and small amount of Si, S, K, Mg, Al, Ca, and Fe, besides Cu with maximum percentage. The samples obtained after the 17th stage contained traces of P too. The Cu content was found to be increased in the final stage of the TP sample when compared to the initial stage. In order to trace the origin of these elements, EDAX spectra of lyophilized plant samples were also recorded. The lyophilized samples of Aristolochia and Alangium showed the presence of Na, K, Ca, Mg, Al, Si, Cl, and O. Hence, it was evidently determined that the origin of the nutrient trace elements found on the surface of traditional TP obtained at various stages of pudam preparation are from the herbal plants.

### 3.6 Transmission Electron Microscope

**FIGURE 8:**
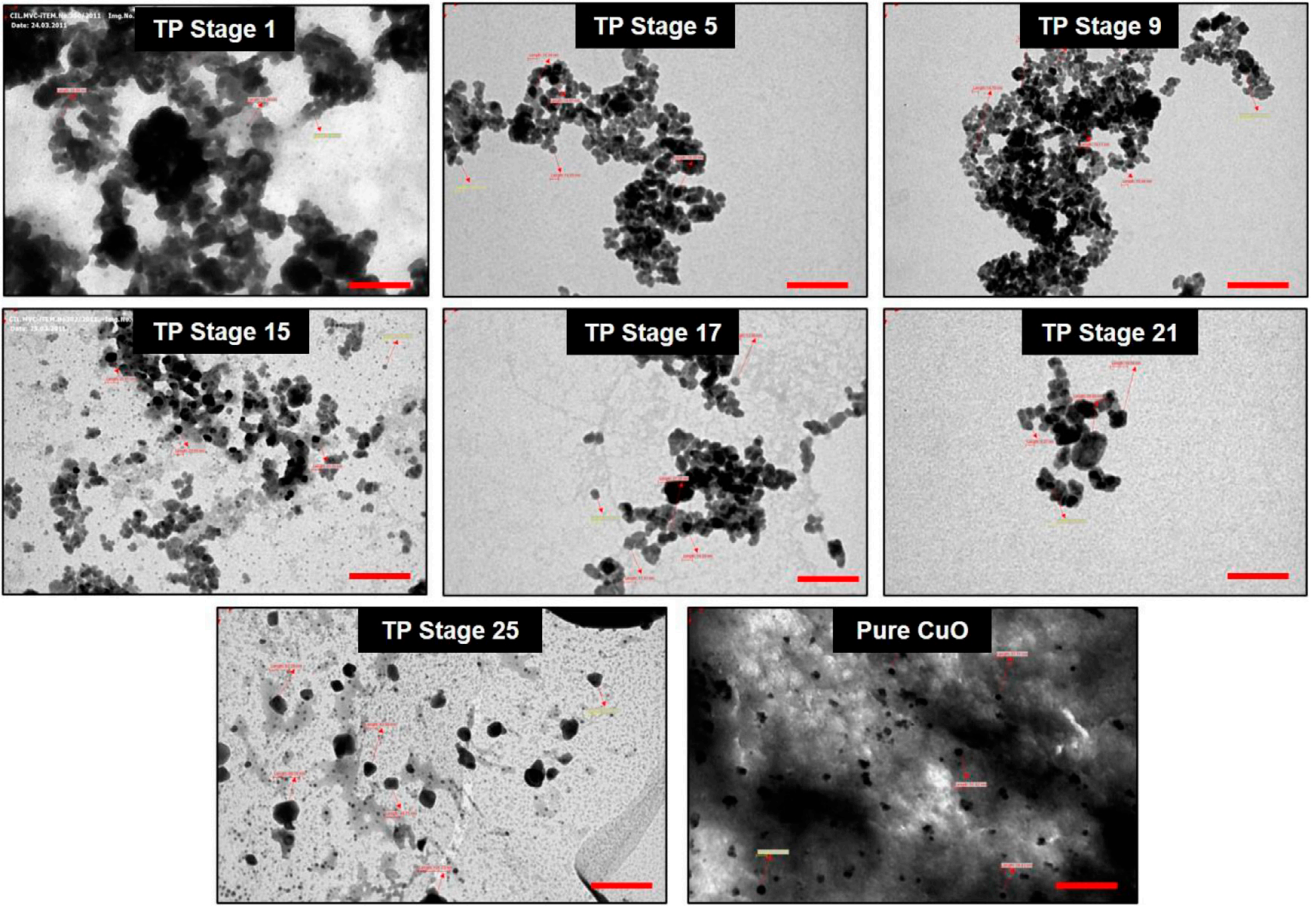
Transmission electron microscopic (TEM) photograph of Thamira parpam particles obtained at various stages of preparation using the traditional ancient method. Scale bar indicates 200 nm.

The particle size analysis of the TP sample also was performed with TEM, and the result is shown in Figure 8. The photomicrograph of bulk particle showed a wide distribution of size in the sample (TP Stage 9). We also observed that the particles were irregular in shape and present in the aggregated form which in the subsequent stages of preparation decreased. The sample obtained in the 25th stage was well-separated, and the particle size was in the range of 10–50 nm with much narrower distribution of sizes. The TEM results confirm the presence of significant number of nanosized particles in the sample.

### 3.7 Toxicological Evaluation Studies

#### 3.7.1 Acute Toxicity

The results indicated that the acute toxicological evaluation of TP via an oral route, at a single dose of 2000 mg/kg b wt. did not produce any signs of toxicity, such as ataxia, convulsion, exophthalmos, lacrimation, oral/nasal, gait, piloerection, polyurea, and rough coat or death, in female SD rats during the period of 14 days of (Table 1). No mortality or morbidity was also observed related to the single-dose TP treatment. No gross lesions were observed during necropsy. Based on these observations, the LD_50_ value for the TP was found to be greater than 2000 mg/kg b wt.

**TABLE 1:** Effect of *in-house* Thamira parpam on clinical signs (Acute and Sub-acute).

Clinical signs	Treatment									
	Normal		Vehicle Control		Low		Mid		High	
	Male	Female	Male	Female	Male	Female	Male	Female	Male	Female
Ataxia	X	X	X	X	X	X	X	X	X	X
Convulsion	X	X	X	X	X	X	X	X	X	X
Exophthalmos	X	X	X	X	X	X	X	X	X	X
Lacrimation	X	X	X	X	X	X	X	X	X	X
Oral/nasal discharges	X	X	X	X	X	X	X	X	X	X
Gait	X	X	X	X	X	X	X	X	X	X
Piloerection	X	X	X	X	X	X	X	X	X	X
Polyuria	X	X	X	X	X	X	X	X	X	X
Rough coat	X	X	X	X	X	X	X	X	X	X

X—No sign/√ - Present.

#### 3.7.2 Sub-Acute Toxicity

No signs of clinical toxicity (Table 1) or mortality were observed in any of the experimental animals during the 28 repeated days of treatment with Thamira Parpam *via* the oral route at doses of 10, 20, and 40 mg/kg b wt. Functional observations also showed no significant changes.

### 3.8 Body Weight, Feed, and Water Intake

**FIGURE 9:**
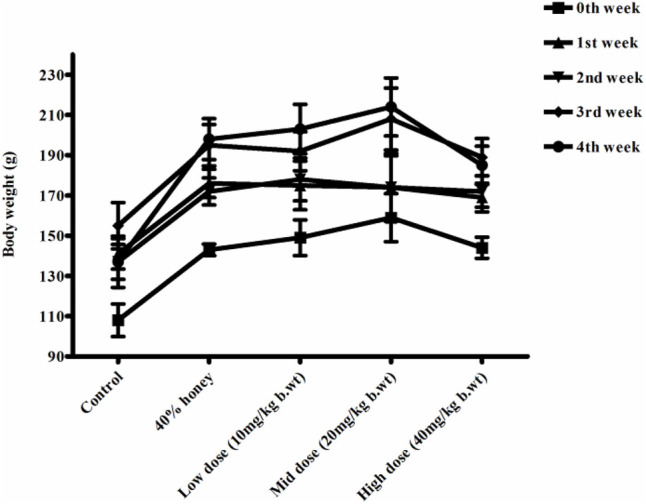
Weekly body weight changes in normal, vehicle, and 28 repeated oral dose of Thamira parpam–treated male SD rats.

**FIGURE 10:**
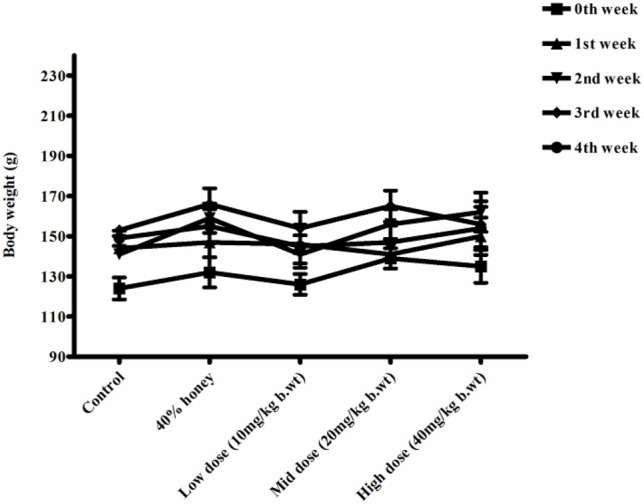
Weekly body weight changes in normal, vehicle, and 28 repeated oral dose of Thamira parpam–treated female SD rats.

No significant differences were found between the initial and final body weight of the male and female rats treated with TP compared to that of the vehicle control rats (Figures 9, 10). No treatment-related changes were observed in the weekly cumulative food and water consumption (Tables 2, 3).

**TABLE 2:** Effect of *in-house* Thamira parpam on feed intake.

Treatment	Sex	Feed intake (g)			
		Day 7	Day 14	Day 21	Day 28
Normal	M(n = 5)	74.46 ± 0.97	57.15 ± 0.87	62.00 ± 0.69	45.15 ± 1.25
	F (n = 5)	75.83 ± 1.12	55.63 ± 0.94	55.02 ± 0.61	65.96 ± 1.49
Vehicle control (40% Honey)	M(n = 5)	91.58 ± 0.54	78.05 ± 1.11	78.72 ± 1.34	78.01 ± 0.36
	F (n = 5)	65.09 ± 1.11	79.59 ± 0.65	64.58 ± 0.96	63.10 ± 0.63
Low dose (10 mg/kg)	M(n = 5)	101.54 ± 0.92	73.90 ± 0.91	79.39 ± 0.56	87.15 ± 1.15
	F (n = 5)	72.80 ± 0.56	79.18 ± 0.69	62.09 ± 0.73	65.15 ± 0.24
Mid dose (20 mg/kg)	M(n = 5)	100.90 ± 0.59	70.20 ± 0.45	68.70 ± 1.06	88.93 ± 0.89
	F (n = 5)	79.61 ± 0.27	72.72 ± 0.99	53.27 ± 0.46	60.88 ± 0.60
High dose (40 mg/kg)	M(n = 5)	108.80 ± 0.90	77.94 ± 1.61	66.35 ± 0.50	75.46 ± 0.87
	F (n = 5)	85.37 ± 0.55	84.81 ± 0.81	62.38 ± 0.57	67.67 ± 0.70

All values expressed as mean±SEM. ***, ** & *p value of<0.001, 0.01, and 0.05, respectively, compared to normal and ###. ## & #*p* value of <0.001, 0.01, and 0.05 compared to vehicle control (40% honey)

**TABLE 3:** Effect of *in-house* Thamira parpam on water intake.

Treatment	Sex	Water intake (ml)			
		Day 7	Day 14	Day 21	Day 28
Normal	M(n = 5)	106.61 ± 1.61	78.94 ± 0.92	88.39 ± 0.73	60.71 ± 2.31
	F (n = 5)	114.92 ± 1.68	104.86 ± 1.30	101.91 ± 0.74	123.74 ± 1.09
Vehicle control (40% Honey)	M(n = 5)	111.76 ± 2.42	138.64 ± 1.04	129.96 ± 0.83	148.45 ± 0.84
	F (n = 5)	127.88 ± 0.65	144.85 ± 0.95	132.24 ± 0.67	97.01 ± 1.40
Low dose 10 mg/kg	M(n = 5)	123.56 ± 1.30	143.79 ± 1.74	96.64 ± 0.96	165.00 ± 1.43
	F (n = 5)	88.08 ± 0.42	112.74 ± 0.91	109.00 ± 1.22	113.41 ± 1.11
Mid dose 20 mg/kg	M(n = 5)	165.45 ± 1.49	145.44 ± 0.91	155.48 ± 1.93	177.05 ± 1.31
	F (n = 5)	115.58 ± 1.34	123.02 ± 0.75	96.39 ± 0.98	68.31 ± 1.32
High dose 40 mg/kg	M(n = 5)	158.05 ± 1.00	142.89 ± 0.67	161.36 ± 0.27	134.56 ± 1.19
	F (n = 5)	127.80 ± 0.73	157.91 ± 0.74	91.64 ± 1.12	119.10 ± 0.52

All values expressed as Mean ± SEM. ***, ** & *p value of <0.001, 0.01, and 0.05, respectively, compared to normal and ###. ## & #*p* value of <0.001, 0.01, and 0.05 compared to vehicle control (40% honey).

### 3.9 Hematological Analysis

Table 4 showed the hematological profile of rats of both sexes administered with TP for 28 consecutive days. No significant treatment-related changes were observed in all the three doses of TP when compared to the vehicle control group. This suggests that TP did not induce any toxicity on the hematopoietic system of the body.

**TABLE 4:** Effect of *in-house* Thamira parpam on 28th day hematological profile.

Treatment	Sex	Hematological parameters								
		WBC (10*3/uL)	RBC (10*6/uL)	Hb (g/dl)	HCT (%)	PLT (10*3/uL)	PCT (%)	MCH (pg)	Rdw-Cv (%)	MCV (fL)
Normal	M(n = 5)	6.28 ± 1.89	8.13 ± 1.19	12.94 ± 2.93	27.10 ± 5.59	165.40 ± 43.07	1.05 ± 0.61	37.40 ± 5.87	15.60 ± 3.56	46.94 ± 1.35
	F(n = 5)	6.46 ± 0.90	7.93 ± 0.42	12.67 ± 0.47	25.72 ± 1.85	164.35 ± 8.55	0.07 ± 0.01	29.96 ± 2.60	15.94 ± 0.39	42.24 ± 0.72
Vehicle control (40% honey)	M(n = 5)	6.60 ± 1.02	8.04 ± 0.88	12.96 ± 0.40	25.06 ± 4.34	156.40 ± 6.82	0.64 ± 0.19	36.04 ± 7.96	15.30 ± 0.09	46.72 ± 0.91
	F(n = 5)	6.36 ± 0.70	7.73 ± 0.49	13.14 ± 0.09	27.70 ± 2.31	157.60 ± 14.42	0.47 ± 0.07	22.38 ± 2.01	14.08 ± 0.31	46.00 ± 0.39
Low dose (10 mg/kg b wt)	M(n = 5)	5.82 ± 0.73	8.66 ± 0.33	12.46 ± 0.65	25.14 ± 1.80	159.80 ± 16.23	0.38 ± 0.14	32.06 ± 1.15	15.18 ± 0.70	46.36 ± 0.72
	F(n = 5)	5.54 ± 0.61	8.38 ± 0.98	12.18 ± 0.77	25.24 ± 2.03	155.00 ± 7.60	0.28 ± 0.12	24.86 ± 1.96	14.84 ± 0.27	45.60 ± 0.51
Mid dose (20 mg/kg b wt)	M(n = 5)	6.26 ± 0.77	8.74 ± 1.11	12.38 ± 0.49	25.26 ± 1.75	157.20 ± 11.09	1.10 ± 0.32	34.54 ± 8.00	17.48 ± 0.96	44.66 ± 1.15
	F(n = 5)	6.84 ± 1.30	8.46 ± 0.11	12.66 ± 0.22	29.04 ± 0.34	141.40 ± 10.88	0.21 ± 0.08	20.30 ± 0.36	15.12 ± 0.49	46.70 ± 0.42
High dose (40 mg/kg b wt)	M(n = 5)	6.56 ± 1.19	8.31 ± 1.08	12.56 ± 0.40	26.00 ± 3.04	168.40 ± 7.83	0.35 ± 0.13	34.78 ± 6.31	15.52 ± 0.90	45.68 ± 0.94
	F(n = 5)	6.80 ± 1.04	8.45 ± 0.48	12.52 ± 0.32	28.22 ± 0.91	151.00 ± 3.67	0.55 ± 0.19	24.58 ± 2.53	14.58 ± 0.25	45.72 ± 0.51

All values expressed as Mean ± SEM. ***, ** & *p value of <0.001, 0.0,1 and 0.05, respectively, compared to normal and ###. ## & #*p* value of <0.001, 0.01, and 0.05 compared to vehicle control (40% honey).

### 3.10 Biochemical Analysis

The female low-dose group (10 mg/kg b wt) showed an elevated glucose level compared to its corresponding vehicle control (40% honey) which was found to be statistically significant (*p* < 0.05), but may not be of clinical relevance since the value was within normal range. Similarly, slight elevation (*p* < 0.05) in the triglycerides level of the vehicle control (40% honey) female group was observed compared to the vehicle control. No significant changes were observed in total cholesterol, protein, albumin, and globulin with TP administration for 28 repeated days (Tables 5, 6). The urea, creatinine, and total bilirubin levels of all the three doses of TP treatment in both the sexes remained unaffected and were well within the normal range following 28 days of administration of TP.

**TABLE 5:** Effect of *in-house* Thamira parpam on 28th day: biochemical parameters (Metabolites).

Treatment	Sex	Plasma metabolites			
		Glucose (mg/dl)	Triglycerides (mg/dl)	Cholesterol (mg/dl)	Bilirubin (mg/dl)
Normal	M(n = 5)	81.04 ± 5.50	76.93 ± 1.85	217.03 ± 3.81	0.31 ± 0.19
	F(n = 5)	86.66 ± 2.33	71.18 ± 4.89	222.06 ± 5.50	0.04 ± 0.02
Vehicle control (40% Honey)	M(n = 5)	82.84 ± 2.13	76.99 ± 3.00	223.82 ± 4.00	0.15 ± 0.06
	F(n = 5)	80.54 ± 5.76	81.08 ± 5.94	242.40 ± 8.53	0.03 ± 0.03
Low-dose (10 mg/kg b wt)	M(n = 5)	86.88 ± 3.78	68.20 ± 4.55	224.42 ± 6.36	0.12 ± 0.09
	F(n = 5)	96.04 ± 4.77^#^	66.92 ± 2.27	216.46 ± 13.36	0.00 ± 0.00
Mid-dose (20 mg/kg b wt)	M(n = 5)	86.14 ± 5.95	78.44 ± 5.91	221.36 ± 5.10	0.00 ± 0.00
	F(n = 5)	82.66 ± 6.57	78.23 ± 3.38	240.74 ± 12.17	0.22 ± 0.17
High-dose (40 mg/kg b wt)	M(n = 5)	92.81 ± 7.24	75.27 ± 3.88	218.92 ± 17.39	0.13 ± 0.06
	F(n = 5)	93.57 ± 7.97	73.28 ± 2.20	261.34 ± 9.17	0.04 ± 0.04

All values expressed as Mean ± SEM. ***, ** & *p value of <0.001, 0.01, and 0.05 respectively compared to normal and ###. ## & #*p* value of <0.001, 0.01, and 0.05 compared to vehicle control (40% honey).

**TABLE 6:** Effect of *in-house* Thamira parpam on 28th day: Biochemical Parameters (Metabolites).

Treatment	Sex	Plasma metabolites				
		Urea (m g/dl)	Creatinine (mg/dl)	Protein (g/dl)	Albumin (g/dl)	Globulin (g/dl)
Normal	M(n = 5)	40.45 ± 1.42	0.70 ± 0.03	4.75 ± 0.20	2.06 ± 0.15	2.69 ± 0.15
	F(n = 5)	39.35 ± 1.48	0.51 ± 0.03	4.73 ± 0.39	2.32 ± 0.11	2.41 ± 0.60
Vehicle control (40% Honey)	M(n = 5)	39.89 ± 2.97	0.64 ± 0.011	4.70 ± 0.22	2.25 ± 0.17	2.45 ± 0.35
	F(n = 5)	38.62 ± 3.66	0.60 ± 0.03	5.11 ± 0.26	2.34 ± 0.20	2.77 ± 0.36
Low dose (10 mg/kg b wt)	M(n = 5)	36.88 ± 3.54	0.68 ± 0.11	5.07 ± 0.27	1.92 ± 0.18	3.15 ± 0.37
	F(n = 5)	34.16 ± 2.33	0.62 ± 0.04	5.20 ± 0.31	2.59 ± 0.16	2.61 ± 0.45
Mid dose (20 mg/kg b wt)	M(n = 5)	34.72 ± 4.12	0.54 ± 0.05	4.55 ± 0.17	2.25 ± 0.09	2.29 ± 0.21
	F(n = 5)	34.62 ± 3.57	0.66 ± 0.08	4.85 ± 0.23	2.62 ± 0.23	2.23 ± 0.99
High dose (40 mg/kg b wt)	M(n = 5)	39.69 ± 9.36	0.68 ± 0.06	5.20 ± 0.11	2.47 ± 0.06	2.73 ± 0.16
	F(n = 5)	35.50 ± 1.75	0.52 ± 0.02	5.10 ± 0.30	3.05 ± 0.07	2.15 ± 0.31

All values expressed as Mean ± SEM. ***, ** & *p value of <0.001, 0.01, and 0.05, respectively, compared to normal and ###, ## & #*p* value of <0.001, 0.01, and 0.05 compared to vehicle control (40% honey).

There were no significant treatment-related changes in the liver function tests, namely, SGOT, SGPT, ALP, and ACP in comparison to the vehicle control group (Table 7). The mid-dose and high-dose female groups exhibited statistically significant (*p* < 0.05) decrease in the γ-glutamyl transferase level, whereas the other groups did not exhibit any treatment-related changes.

**TABLE 7:** Effect of *in—house* Thamira parpam on 28th day: biochemical parameters (Enzymes).

Treatment	Sex	Plasma enzymes				
		SGOT (U/L)	SGPT (U/L)	ALP (U/L)	ACP (U/L)	GGT (U/L)
Normal	M(n = 5)	149.29 ± 5.40	59.67 ± 3.98	604.45 ± 7.03	79.73 ± 3.02	23.38 ± 1.04
	F(n = 5)	122.60 ± 5.07	57.63 ± 3.54	563.81 ± 13.34	70.66 ± 2.31	20.39 ± 0.93
Vehicle control (40% Honey)	M(n = 5)	144.70 ± 3.83	51.40 ± 2.80	569.20 ± 12.90	70.32 ± 2.41	21.13 ± 0.99
	F(n = 5)	128.28 ± 7.34	53.71 ± 8.00	529.06 ± 32.60	69.50 ± 5.44	20.26 ± 0.96
Low dose (10 mg/kg b wt)	M(n = 5)	142.46 ± 6.97	58.35 ± 5.61	605.80 ± 11.62	70.14 ± 7.38	18.86 ± 1.12
	F(n = 5)	120.16 ± 5.23	55.58 ± 4.06	589.78 ± 14.41	68.07 ± 6.96	19.26 ± 0.88
Mid dose (20 mg/kg b wt)	M(n = 5)	138.36 ± 4.27	55.75 ± 3.49	567.40 ± 5.50	71.39 ± 2.94	17.04 ± 0.92
	F(n = 5)	125.12 ± 8.67	54.68 ± 7.49	549.02 ± 34.97	68.04 ± 3.45	16.63 ± 0.92^#^
High dose (40 mg/kg b wt)	M(n = 5)	149.38 ± 21.96	67.93 ± 5.67	585.18 ± 51.93	74.53 ± 2.71	17.00 ± 1.23
	F(n = 5)	129.10 ± 5.39	59.99 ± 10.49	568.96 ± 40.98	68.78 ± 4.92	14.29 ± 0.50^#^

All values expressed as Mean ± SEM. ***, ** & *p value of <0.001, 0.01, and 0.05, respectively, compared to normal and ###. ## & #*p* value of <0.001, 0.01, and 0.05 compared to vehicle control (40% honey).

### 3.11 Organ Weight and Histopathology

**FIGURE 11:**
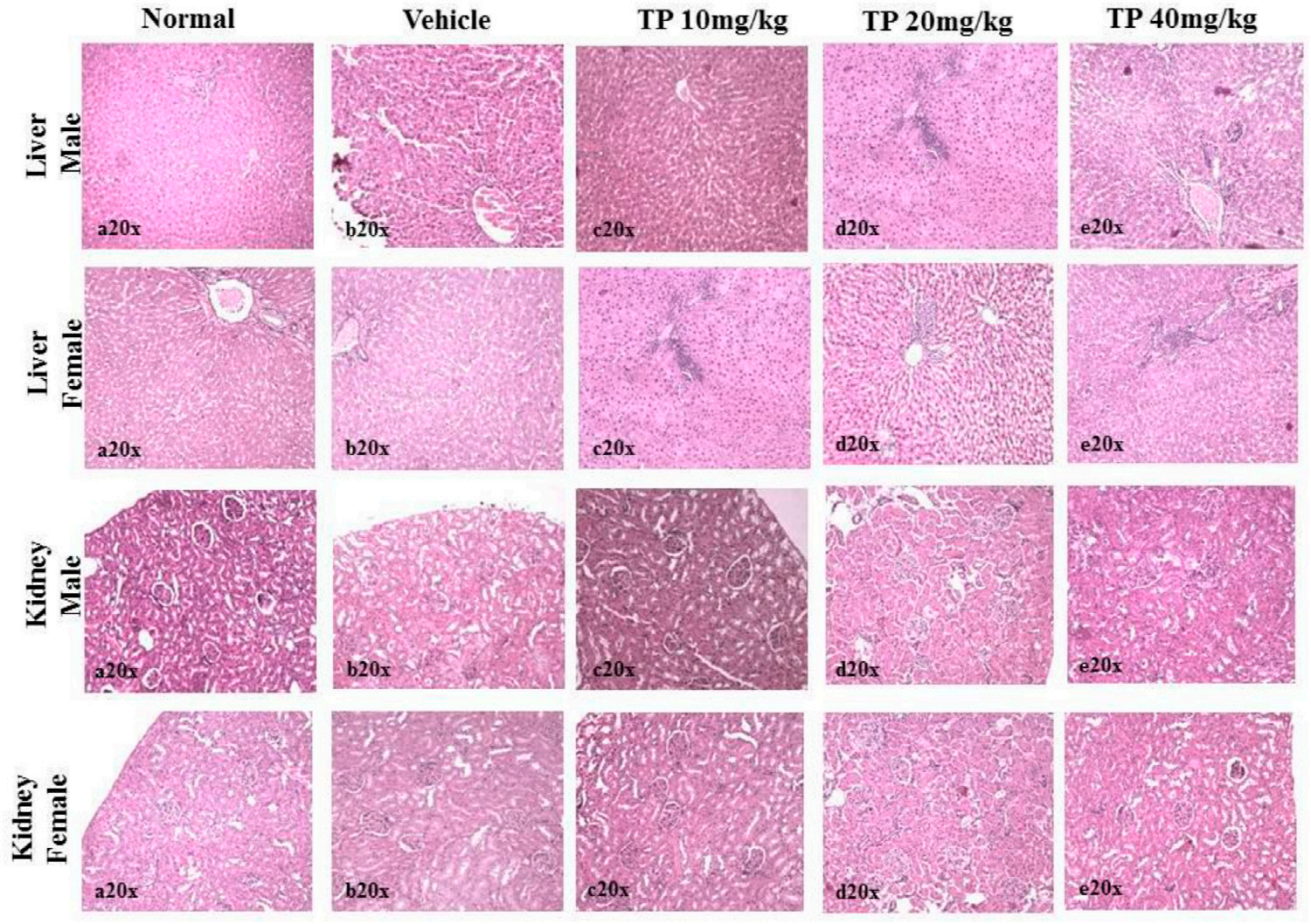
Histopathological analysis of the liver showing no normal hepatocytes in control, vehicle, and low-dose TP-treated groups of both sexes. Mild portal inflammation observed in the mid-and high-dose TP-treated groups of male and female rats. Histopathological analysis of kidney tissues showed normal architecture of nephrons in the male and female SD rats after 28 days treatment of Thamira parpam.

**FIGURE 12:**
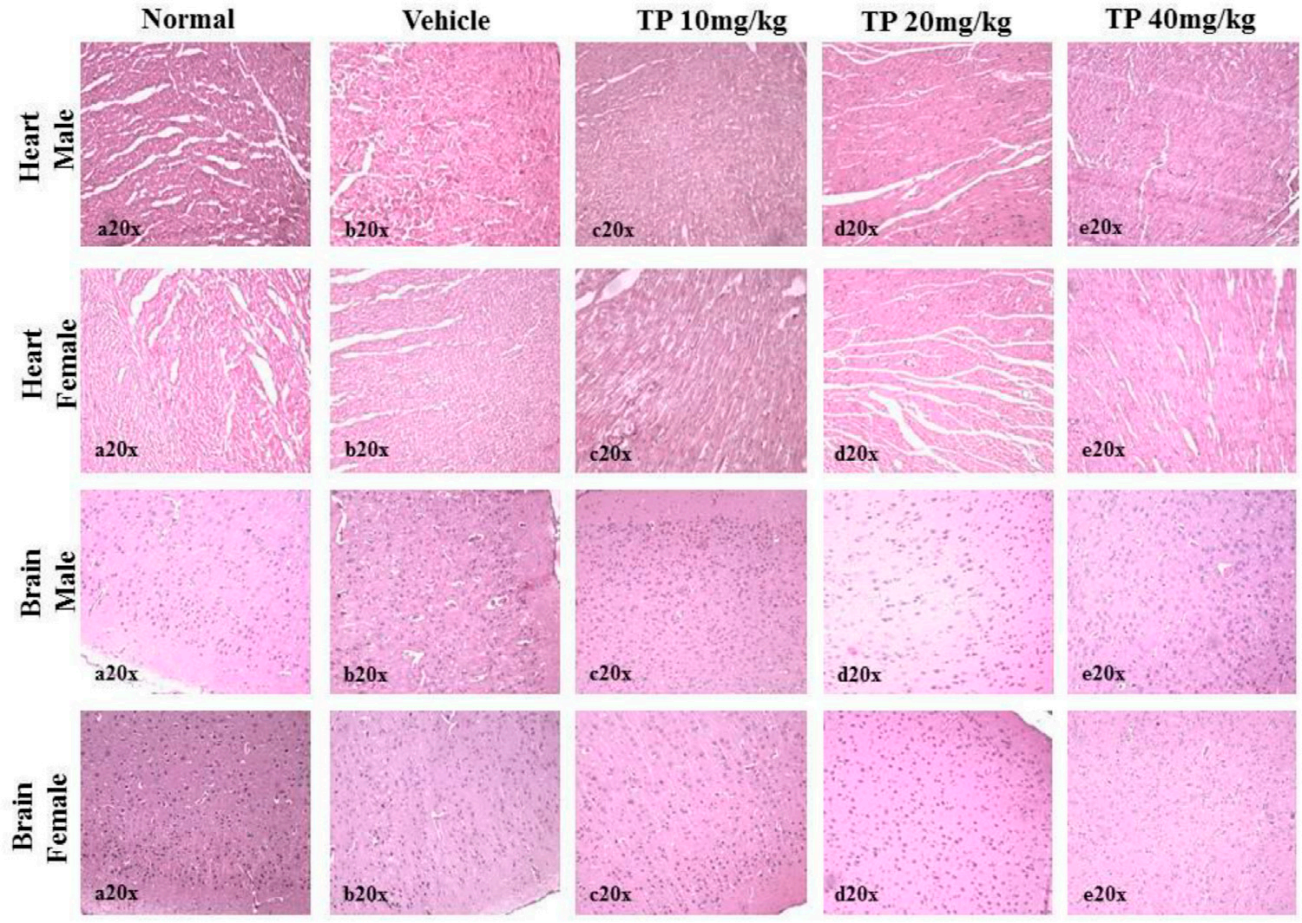
Histopathological analysis of heart and brain tissues showing no abnormalities in the male and female SD rats after 28 days treatment of Thamira Parpam.

**FIGURE 13:**
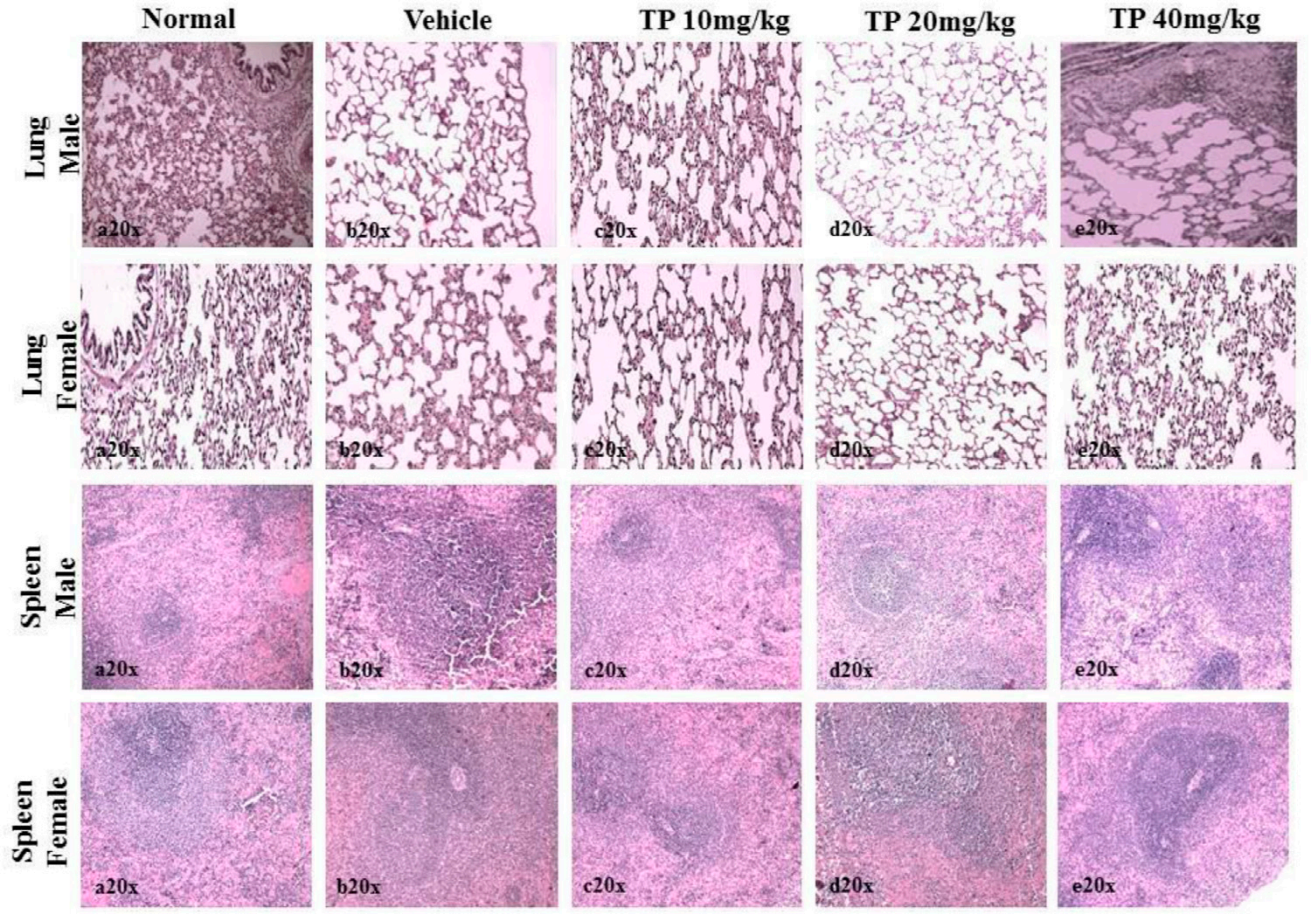
Histopathological analysis of lung and spleen tissue showing no abnormalities in the male and female SD rats after 28-day treatment of Thamira Parpam.

**FIGURE 14:**
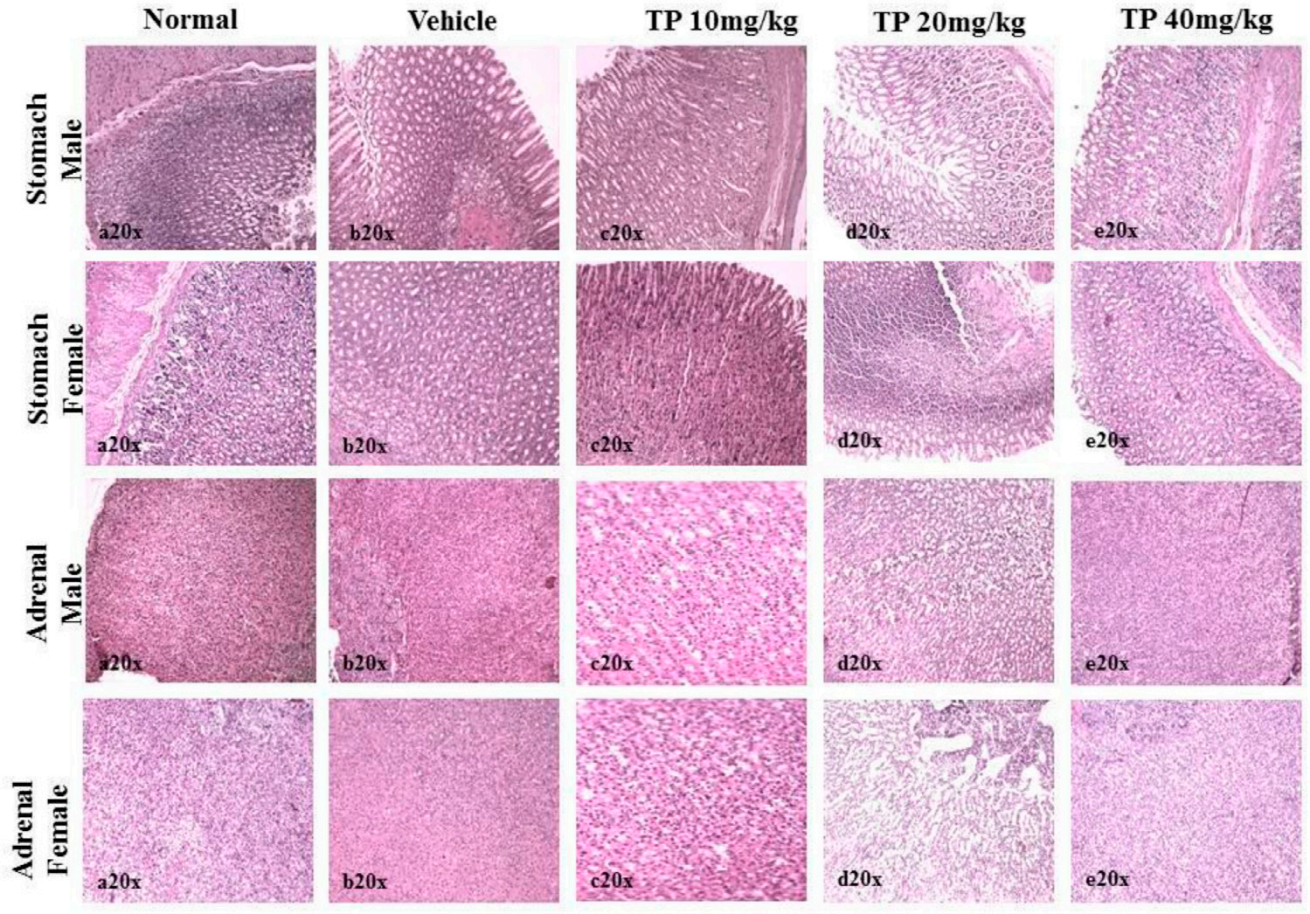
Histopathological analysis of stomach and adrenals showing no ulceration and abnormalities, respectively, in the male and female SD rats after 28-day treatment of Thamira parpam.

**FIGURE 15:**
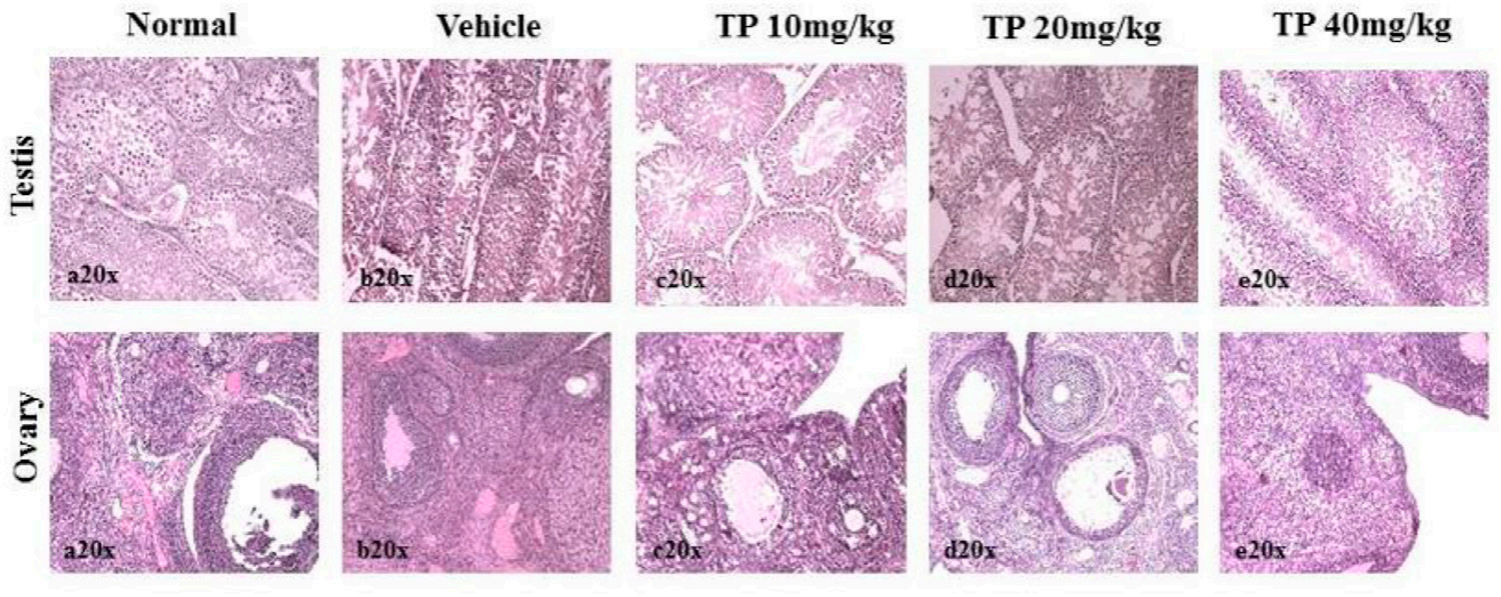
Histopathological analysis of sex organs showing normal architecture in the male and female SD rats after 28-day treatment of Thamira Parpam.

No significant changes were observed in the organ weights of any of the groups except for the weight of the liver in mid- and high-dose groups of both sexes while comparing to the vehicle control group (Table 8). Mild inflammation was observed in the mid- and high-dose groups of TPs of both sexes when compared to the vehicle control group (Figure 11). Despite observed mild inflammation in the liver tissue, these changes were considered impulsive as minor deviations are relatively expected on long-term administration of metal-based preparations. No pathological abnormalities were observed in the other tissues (Figure 12–15).

**TABLE 8:** Effect of *in-house* Thamira parpam on Organ weight.

Treatment	Sex	Organ Weight(g)								
		Brain	Eyes	Heart	Lung	Liver	Spleen	Kidney	Adrenal	Sex organs
Normal	M(n = 5)	1.05 ± 0.43	0.19 ± 0.08	0.42 ± 0.17	0.90 ± 0.41	7.27 ± 1.35	0.29 ± 0.13	0.86 ± 0.35	0.02 ± 0.01	1.03 ± 0.49
	F (n = 5)	1.43 ± 0.36	0.23 ± 0.06	0.60 ± 0.16	1.26 ± 0.32	5.60 ± 1.46	0.52 ± 0.14	1.24 ± 0.31	0.04 ± 0.01	0.07 ± 0.03
Vehicle control (40% Honey)	M(n = 5)	2.02 ± 0.04	0.34 ± 0.04	0.97 ± 0.02	2.00 ± 0.19	9.36 ± 0.33	0.91 ± 0.07	1.87 ± 0.15	0.06 ± 0.01	2.33 ± 0.16
	F (n = 5)	1.80 ± 0.04	0.38 ± 0.04	0.66 ± 0.04	1.46 ± 0.22	6.64 ± 0.41	0.58 ± 0.07	1.47 ± 0.08	0.05 ± 0.01	0.09 ± 0.01
Low dose (10 mg/kg b wt)	M(n = 5)	1.88 ± 0.04	0.33 ± 0.04	0.89 ± 0.06	1.54 ± 0.11	6.98 ± 0.38	0.77 ± 0.08	1.83 ± 0.13	0.06 ± 0.01	2.56 ± 0.15
	F (n = 5)	1.79 ± 0.07	0.37 ± 0.04	0.63 ± 0.03	1.82 ± 0.55	6.39 ± 0.52	0.64 ± 0.07	1.49 ± 0.07	0.05 ± 0.01	0.08 ± 0.01
Mid dose (20 mg/kg b wt)	M(n = 5)	1.91 ± 0.07	0.32 ± 0.01	0.97 ± 0.04	1.81 ± 0.24	8.78 ± 0.44	0.97 ± 0.09	1.98 ± 0.05	0.05 ± 0.00	2.42 ± 0.24
	F (n = 5)	1.87 ± 0.05	0.37 ± 0.04	0.76 ± 0.06	1.35 ± 0.14	5.70 ± 0.62	0.67 ± 0.05	1.44 ± 0.07	0.07 ± 0.02	0.11 ± 0.03
High dose (40 mg/kg b wt)	M(n = 5)	1.95 ± 0.05	0.36 ± 0.01	0.85 ± 0.03	1.66 ± 0.10	7.46 ± 0.52	0.83 ± 0.08	1.83 ± 0.05	0.05 ± 0.01	2.25 ± 0.44
	F (n = 5)	1.83 ± 0.07	0.40 ± 0.04	0.70 ± 0.04	1.41 ± 0.28	7.02 ± 0.83	0.64 ± 0.05	1.62 ± 0.09	0.08 ± 0.02	0.11 ± 0.01

All values expressed as Mean ± SEM. ***, ** & *p value of <0.001, 0.01, and 0.05, respectively, compared to normal and ###. ## & #*p* value of <0.001, 0.01, and 0.05 compared to vehicle control (40% honey).

## 4 Discussion

Among the several methods of preparing nanomaterials, the top–down synthesis approach which uses a destructive method of reducing the bulk material into nanosized units by various physical and chemical methods is common (Khan et al., 2019). Of late, a number of processes that fabricate nanomaterials using plants, their whole extracts and their phytoconstituents are of interest to many researchers known as “green synthesis” (Gericke and Pinches, 2006). Herein, it is interesting to note that the ancient Siddhars have used this top–down approach that encompasses alternative heating, quenching in plant juices, grinding, and milling followed by calcining by a unique ancient method called the “Pudam” process to prepare TP (Thiagarajan, 1981). Hence evaluating the changes at every stage of TP preparation was worth pondering. In addition, several herbometallic formulations have been reported for their various health benefits in the ancient systems of medicine. Nevertheless, such metal-based drugs are considered toxic by the modern medicine system due to heavy metal load in them. Furthermore, literature on metal-based nanoparticles has demonstrated that the particle size and their toxicity are inversely proportional; smaller the size more the toxicity (Schrand et al., 2010; Luo et al., 2015). Other characteristics which influence the toxicity of metal-based nanoparticles encompass charge, shape, solubility, and surface characteristics (Luo et al., 2015). In view of this, we have carried out an intensive study on the preparation method and characterization of TP and also evaluated its toxicity profile.

The physicochemical characterization studies on TP have given some intriguing insights about the traditional Siddha method of preparation “Pudam method.” The TGA results exhibited constant weight loss during the step-by-step preparation of TP from stage 1 to stage 15, after which no weight loss was observed. These weight losses observed at the different stages of TP samples might be due to the removal of adsorbed oxygen or water molecules and any other adsorbed organic moieties in the samples. Furthermore, the presence of extra peaks in the XPS of intermittent TP samples confirmed the reduction in the number of surface oxygen atoms which is because of the presence of mixed oxides Cu_2_O and CuO in it (Bhowmick et al., 2009). The TGA and XPS results substantiates that the repeated cycles of annealing and calcination have led oxygen to chemisorb with copper metals rather than getting adsorbed on the surface (Wadekar, 2005; Wadekar et al., 2006). The presence of oxygen vacancies on the surface of the metal attributes to the enhanced efficacy of metal oxides (Pan et al., 2005; Bhowmick et al., 2009).

Organic moieties cannot withstand higher temperatures, but to our surprise, the EPR data exhibited signals at g ≈ 2, indicating the presence of organic moieties. This could be due to the formation of organometallic complexes in the sample that can sustain high processing temperatures (Singh et al., 2010). The high-intensity XRD lines present in the initial stages of TP revealed crystalline nature, whereas in the final stage, TP crystalline nature was absent. This loss of luster is typical for a better quality parpam as per ancient Indian literature (Chavare et al., 2017). The particle size and morphology of nanosize drugs have been described as key factors in imparting targeted efficacy (Alexiou et al., 2000; Chithrani et al., 2006). Studies have shown that the calcination temperature and its duration during the preparation of herbometallic drugs possess an impact on the morphology (Centi et al., 1997; Borgohain et al., 2001). Hence, the agglomeration of particles which was observed in the SEM analysis might be due to repeated cycles of calcinations involved in preparation. It was also confirmed in TEM analysis that the drug TP was around 50 nm in size, confirming it as a nanoparticle.

**FIGURE 16:**
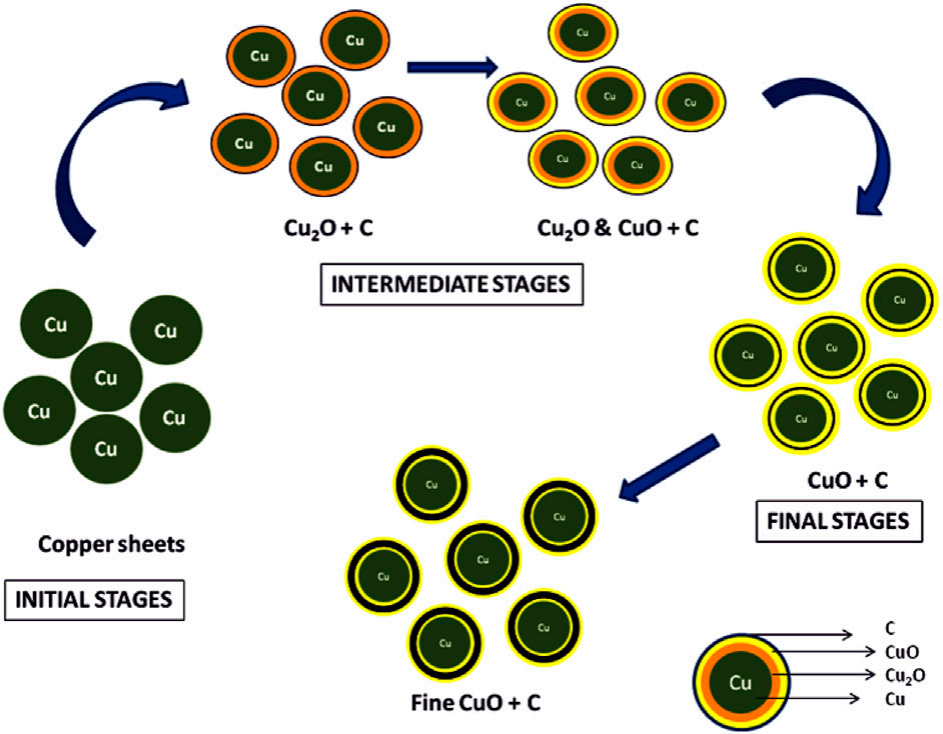
Illustration of stepwise formation of CuO involved in Thamira parpam preparation using the traditional ancient method.

One of the key observations in this investigation was the presence of carbonaceous material on the surface of TP (an illustration has been depicted in Figure 16. The source of the carbonaceous coating might be from the organic moieties (viz., hydrocarbons) derived from the plant juices of Aristolochia and Alangium during the pudam process and the effect of repeated calcinations for about 25 cycles above 800°C, which is a traditional claim for the method of preparation (Singh and Rai, 2012). The contributing factors involved in the bonding of the hydrocarbons upon the metal surface are dative bonding, van der Waals force, and hydrophobic interactions. The chemisorption of the carbonaceous matter upon the copper metal is suggestive of the stronger bond formation between electron-donating moieties from the plant juices to an electronegative metal such as copper (Ostrom et al., 2003). Furthermore, the bonding is considered to be stronger with decreased particle size (Phala and van Steen, 2007; Geoffrey, 2012). This further confirms that the metal is not in the free state but in the organo-metallic state, which probably increases the safety of the nanoparticulate TP.

The immune system being a subtle target for toxic compounds was considered to be an imperative manifestation for the determination of toxicity (Mukinda and Syce, 2007). The results of the present study demonstrated no hematological alterations suggesting the nontoxic effect of TP. ALT and ACP are the key enzymes of the liver, the elevation of which is considered to be indicative of liver damage (Witthawaskul et al., 2003; Burger et al., 2005). In addition, GGT is a nonspecific liver canalicular enzyme which increases if there is hepatobiliary injury (Rosalki and Mcintyre, 1999; Mauro et al., 2006). The levels of serum proteins, such as albumin and globulin, also play a critical role as markers for evaluating hepatotoxicity, as all these metabolites are synthesized in hepatocytes (Rasekh et al., 2008). In our study, no significant differences in any of these liver enzymes and liver metabolites were observed, indicating that TP on administering as repeated doses had not caused any liver abnormality or toxicity. The significant alteration in the GGT level of mid-dose and high-dose TP-administered female group was not considered to be of toxicological relevance as there were no associated histopathological deviations. Similarly, the study showed no marked alterations in the levels of creatinine and blood urea nitrogen (BUN) which indirectly manifests the nontoxic effect of TP in maintaining the homeostasis of protein and nonprotein nitrogen in body fluids (Evan, 2008).

The assessment of internal organ weight, in general, is a simple and sensible measure of toxicity after exposure to a toxic substance (American Biogenics Corp, 1988). The present investigation showed no significant differences in the organ weights between any of the study groups. Furthermore, no histological alterations in most of the organs examined suggest the protective effect of TP. Yet, the mid-dose and high-dose groups of both sexes showed a mild portal inflammation in the liver. However, it was not considered to be of toxicological relevance, since no marked elevation in the levels of liver enzymes was observed. Further failure in observing any lesion or damage in the tissues both microscopically and macroscopically in addition to relative organ weight correlated with the plasma enzymes and metabolites profile validated the claim that TP is a safe Siddha medicine for human use. Therefore, it is hypothesized that the traditional Siddha methodology preparation of herbometallic formulation TP has made the copper (II), nanoxide, a nontoxic and a nanocomplex carrier of plant juices potentiating the bioefficacy of TP, probably by acting as a drug-delivery nanomesh. Furthermore, no toxic effect was observed up to a dose level of 20 mg/kg b wt. Hence, the no-observed-adverse-effect level (NOAEL) could be considered as 10 mg/kg b. wt and the lowest observed adverse effect level (LOAEL) might be considered > 20 mg/kg b wt.

## 5 Conclusion

The findings from the study conclude that TP is a carbon-coated copper (II) oxide nanoparticle and the plants used for quenching the raw metal sheets might passivate the toxicity of the metal and act as a capping agent that aids in size reduction of the nanoparticle and inhibit self-aggregation. Furthermore, the metal toxicity could have been subdued because of the carbonaceous coating around the nanoparticle copper (II) oxide, confirming that the drug is safe at a low dose.

## Data Availability

The original contributions presented in the study are included in the article/Supplementary Material, further inquiries can be directed to the corresponding author.
